# T cell Metabolism in Lupus

**DOI:** 10.20900/immunometab20200009

**Published:** 2020-02-10

**Authors:** Milena Vukelic, Michihito Kono, George C. Tsokos

**Affiliations:** 1 Department of Medicine, Beth Israel Deaconess Medical Center, Harvard Medical School, Boston, MA 02115, USA; 2 Department of Rheumatology, Endocrinology and Nephrology, Faculty of Medicine, Hokkaido University, Sapporo 060-0808, Japan

**Keywords:** T cell metabolism, glycolysis, glutaminolysis, fatty acid oxidation, SLE

## Abstract

Abnormal T cell responses are central to the development of autoimmunity and organ damage in systemic lupus erythematosus. Following stimulation, naïve T cells undergo rapid proliferation, differentiation and cytokine production. Since the initial report, approximately two decades ago, that engagement of CD28 enhances glycolysis but PD-1 and CTLA-4 decrease it, significant information has been generated which has linked metabolic reprogramming with the fate of differentiating T cell in health and autoimmunity. Herein we summarize how defects in mitochondrial dysfunction, oxidative stress, glycolysis, glutaminolysis and lipid metabolism contribute to pro-inflammatory T cell responses in systemic lupus erythematosus and discuss how metabolic defects can be exploited therapeutically.

## INTRODUCTION

Systemic lupus erythematosus (SLE) is a chronic autoimmune condition characterized by abnormal T cell responses to self-antigens resulting in multi-organ involvement including skin, kidney and central nervous system [1]. Following the initial report, two decades ago, that engagement of CD28 leads to enhanced glycolysis in T cells [2] plethora of data contributed to our current understanding on how metabolic processes are involved in the control of various aspects of T cell signaling, differentiation and pathogenicity allowing for the development of new therapeutic tools or repurposing of already known drugs for the treatment of patients with SLE [3–5]. Advancements in nuclear magnetic resonance spectroscopy and gas chromatography/mass spectrometry have led to the identification of metabolic biomarkers in SLE [3–6]. Herein, we focus on the most recent understandings of the metabolic abnormalities in T cell subsets in patients with SLE and discuss how metabolic defects can be exploited therapeutically.

## MITOCHONDRIAL DYSFUNCTION AND OXIDATIVE STRESS IN SLE

Increased oxidative stress and altered redox state have been shown to contribute to pathogenesis and tissue damage in patients with SLE by increasing apoptosis, decreasing the clearance of apoptotic material and oxidative modification of numerous biomolecules including DNA and enzymes [7–9]. Reactive oxygen species (ROS) is a group free radical generated during mitochondrial respiration as the result of incomplete reduction of oxygen. Under normal and tightly controlled physiological conditions these molecules play positive role in CD4^+^ T cell signaling and homeostasis such as antigen-specific proliferation, differentiation and cytokine production [10]. Loss of mitochondrial DNA or disruption of mitochondrial complex I or III results in low ROS production and leads to reduced production of interleukin (IL)-2 and IL-4 [11]. In CD4^+^ T cells from healthy people engagement of the costimulatory molecule CD28 leads to rapid upregulation of aerobic glycolysis [2], which is in stark contrast to T cells from patients with SLE which display a chronically activated phenotype, upregulated calcium signaling, enhanced tricarboxylic acid (TCA) cycle activity and dependency on oxidative phosphorylation (OXPHOS) to meet their energetic needs [12]. By shifting away from aerobic glycolysis and pentose phosphate pathway, SLE CD4^+^ T cells eventually exhaust their antioxidant capacity with lower glutathione and NADPH pools [9,13]. At the subcellular level, electron microscopy has revealed extensive mitochondrial remodeling in CD4^+^ T cells isolated from people with SLE with the development of hyperpolarized megamitochondria [14], but with paradoxically decreased ATP production and marked leakage of ROS outside of the organelles [15]. Besides chronic stimulation and reliance on OXPHOS, genetic contributors have been postulated to play a role in the abnormal mitochondrial homeostasis. In humans, a SNP variant of the ATP6 or F0F1-ATPase gene (Complex V) has been associated with the development of SLE [16]. Inhibition of this ATPase leads to mitochondrial hyperpolarization and ATP depletion, features similar to those observed in SLE, but in vivo treatment of MRL/*lpr* mice with Bz-423, an inhibitor of mitochondrial F1F0 ATP synthase, leads to apoptosis of autoreactive CD4^+^ T cells and suppression of glomerulonephritis [17]. The murine lupus susceptibility locus Sle1c2 defines the *Esrrg* gene, which is a known regulator of mitochondrial function, and whose decreased expression in lupus-prone mice contributes to mitochondrial dysfunction with increased ROS leakage, abnormal CD4^+^ T cell activation and increased IFNγ production [18,19].

Under normal conditions, ROS production by mitochondria is needed to trigger signaling through NF-κB, AP1 and NFAT (which bind to the IL-2 promoter) to promote IL-2 production [10,11,20]. High oxidative stress in SLE T cells [21,22], together with the overexpressed serine-threonine protein phosphatase2A (PP2A) leads to T-cell receptor (TCR) rewiring by promoting replacement of CD3ζ with FcεRIγ chain coupled with SYK and decreased DNA mehyltransferase 1 activity [21–23]. In parallel, oxidative stress impairs ERK pathway signaling by decreasing protein kinase C δ (PKCδ) phosphorylation and DNA methyltransferase 1 activity, thus directly leading to hypomethylated status of DNA observed in SLE and overexpression of genes associated with pathogenesis of SLE [23–29]. Additionally, ROS triggers activation of mammalian target of rapamycin (mTOR) which is a sensor of mitochondrial hyper polarization and nutrient status [30,31]. In turn, mTOR signaling is directly involved in maintaining and promoting increased mitochondrial biomass by decreasing mitophagy [32]. In contrast to mTORC2, increased activation of mTORC1 is observed in CD4^+^ T cells obtained from SLE patients and lupus prone mice leading to elevated IL-17, IL-4 producing double negative T cell expansion and regulatory T cell (Treg) depletion [33–35]. Unrestricted mTORC1 signaling leads to severe SLE-related manifestations and this is highlighted in reports of several patients with mutations in tuberous sclerosis complex genes which are known suppressors of mTORC1 signaling [36,37]. Signaling through mTORC1 shifts balance of CD4^+^ T cell polarization away from Treg development and toward Th1 and Th17 phenotype by enhancing glycolysis (in these subsets), activates retinoic acid-related orphan receptor gamma t (RORγt) and signal transducer and activator of transcription 3 (STAT3) phosphorylation [33,34,38]. The activity of mTORC1 in Treg is curbed by PP2A and even though mTORC1 does not influence Foxp3 expression and is necessary for the maintenance of suppressive function by Treg cells [39–42]. The inhibition of mTORC1 with rapamycin leads to Treg cell expansion, contraction of IL-17 producing cells and suppression of STAT3 signaling—all of which represent attractive therapeutic targets in people with SLE [43–45]. In addition, in vitro treatment with rapamycin reduces glycolysis and mitochondrial potential and corrects the replacement of CD3ζ chain on CD4^+^ T cells [46,47]. Moreover, there is complex fine-tuning between mTORC1 and 2 complexes in Treg cells as they transition through various stages of differentiation [39,48].

Germinal center formation depends on the presence of follicular helper T cells (Tfh) which are expanded in people with SLE [49]. There are conflicting results whether Tfh differentiation is independent or not of mTORC1 activity but more indirect evidence has implicated mTORC2 in Tfh cell differentiation [41,42,50]. Treatment with the reducing agent *N-*acetylcysteine proved beneficial in SLE patients and it reversed the expansion of double negative T cells, stimulated Foxp3 expression and decreased dsDNA levels [51] (Table 1). Treatment of triple congenic B6.Sle1.Sle2.Sle3 lupus-prone mice with metformin, the inhibitor of mitochondrial metabolism, corrected abnormal T cell metabolism, reduced IFNγ production and restored the IL-2 production [47]. Similarly, metformin normalized in vitro IFNγ production in CD4^+^ T cells isolated from patients with SLE [52]. Also, combination of metformin and glycolytic inhibitor 2-deoxy-D glucose (2-DG) showed a synergic effect in vivo and decreased serological markers of SLE disease activity and improved nephritis (Table 1).

## GLYCOLYSIS

**Figure 1.**
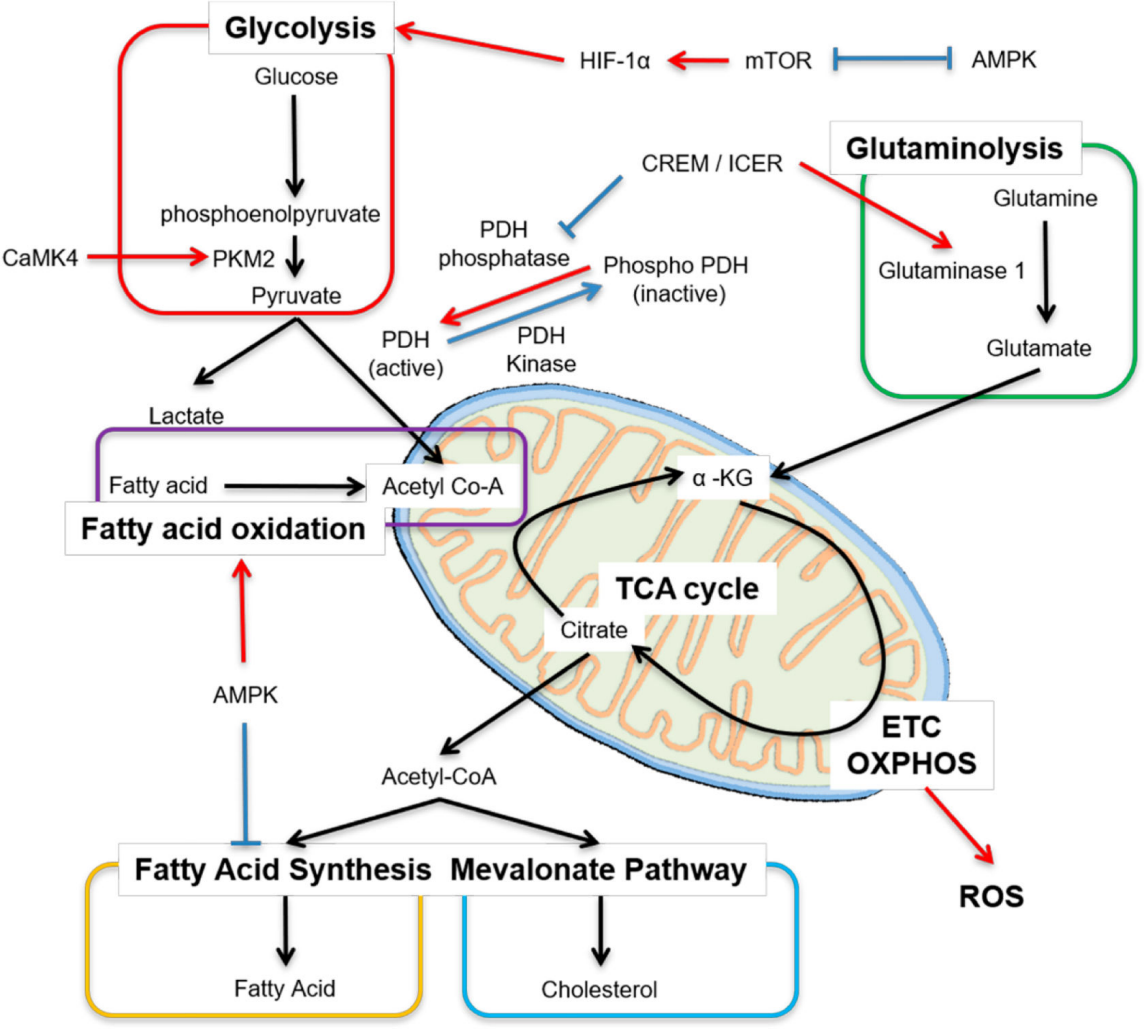
Main metabolic pathways in T cells. Cellular metabolism is controlled by many factors, including transcription factors. Red arrow means “enhance or activate”, whereas blue line means “inhibit or inactivate”. Acetyl Co-A, acetyl coenzyme A; mTOR, mammalian target of rapamycin; AMPK, adenosine monophosphate activated protein kinase; HIF-1α, hypoxia inducible factor 1 alpha; PKM2, pyruvate kinase muscle isozyme 2; CaMK4, calcium/calmodulin–dependent protein kinase IV; PDH, pyruvate dehydrogenase; ICER, inducible cAMP early repressor; α-KG, α-ketoglutarate; ETC, electron transport chain; OXPHOS, oxidative phosphorylation; ROS, reactive oxygen species.

Generation of adenosine triphosphate (ATP) in T cells is essential for their survival, activation, differentiation and effector functions. There is marked diversity between T cells subsets in regard to which metabolic pathway dominates the production of energy [53]. Whether an activated naïve cell will differentiate into effector, regulatory or memory T cell depends, in part, not just on the cytokine milieu but also on metabolic reprogramming [54–56]. At rest, both naïve CD4^+^ and CD8^+^ T cells fulfill their low metabolic demands by utilizing low rates of OXPHOS [57,58]. Somewhat similar metabolic needs are found in Treg cells and memory CD4^+^ T cells that predominantly rely on fatty acid oxidation (FAO) and OXPHOS [59–61] for the production of energy. In contrast to this, differentiated effector CD4^+^ cells prefer glutaminolysis, rapid glycolysis and fatty acid synthesis [59,62] (Figure 1).

Upon activation, naïve T cells rapidly shift metabolism towards aerobic glycolysis with large glucose consumption [58,63]. From the efficiency standpoint oxidative glycolysis is less efficient than TCA cycle coupled to OXPHOS, but serves as a means to engage pentose phosphate pathway (PPP) to generate nucleotides, amino acids, lipids and NADPH to support an increase in the levels of antioxidants in the cell [64,65]. Pyruvate is the end product of glycolysis, and at rest, it is more likely to be converted to lactate rather than to enter the TCA cycle as acetyl coenzyme A (acetyl-CoA) [66]. End products of TCA cycle are NADH, FADH_2_ and amino acids. NADH enters OXPHOS on the inner mitochondrial membrane to generate maximum ATPs. This process is prerequisite for Th1 and Th17 differentiation [67]. Once CD4^+^ T cells are activated, the engagement of TCR and co-stimulatory receptors leads to the rapid upregulation of the glucose transporter Glut1 via PI3K-Akt signaling (that can activate mTOR) and upregulation of key downstream enzymes via hypoxia-inducible factor (HIF)-1α and Myc [2,64] (Figure 1). The opposite occurs with the engagement of cytotoxic T lymphocyte–associated protein 4 (CTLA-4) and programmed death 1 (PD-1) [2,68].

Several metabolic abnormalities have been observed in SLE T cells. Chronic antigenic stimulation leads to increased OXPHOS as measured by the oxygen consumption which can be replicated in healthy cells following repetitive antigen stimulation or in T cells lacking HIF-1α [12,69,70]. As discussed above, in SLE T cells OXPHOS fails to generate sufficient ATP compared to healthy T cells despite having enlarged mitochondrial biomass. Therefore, enhanced secondary glycolysis is observed in SLE [71]. Overexpression of Glut1 in murine T cells results in the development of lupus-like disease in older mice and selective accumulation of effector and follicular T cells [72]. More recently, Glut1 overexpression was found in effector memory CD4^+^ T cells in people with active and inactive SLE [73]. Increased Glut1 expression can be reversed by inhibiting the T cell restricted serine/threonine kinase, calcium/calmodulin–dependent protein kinase IV (CaMK4) which is overexpressed in SLE T cells [73,74]. Pharmacological inhibition or genetic deletion of CaMK4 decreases glycolysis and ameliorates disease activity in MRL/*lpr* mice [75–77]. CaMK4 activates AKT/mTOR pathway but is also found to promote glycolysis by binding and augmenting the activity of pyruvate kinase M2, the final rate-limiting enzyme in glycolysis, underlying autoimmunity associated with Th17 in SLE [78,79]. A distinct feature of Th17 cells, which are exaggerated in patients with SLE, is the overexpression of HIF-1α and reduced pyruvate dehydrogenase (PDH) activity that triggers metabolic shift leading to enhanced pyruvate to lactate production and decreased pyruvate to acetyl-CoA [62,80] (Figure 1). The enzymatic activity of PDH is inhibited in Th17 cells to promote conversion of pyruvate to lactate by promoting the activity of PDH kinase, which phosphorylates PDH (active form) to phospho-PDH (inactive form) [62]. On the other hand, PDH phosphatase makes PDH active (Figure 1) [80]. The cAMP response element modulator (CREM) moderates the transcription of cAMP-responsible genes [81]. CREM splice variants CREMα and inducible cAMP early repressor (ICER) are increased in Th17 cells and more so in people with SLE [82]. ICER binds the cAMP-response element (CRE) of PDH phosphatase catalytic subunit 2 (*Pdp2*) promoter, suppresses the *Pdp2* gene expression and reduces PDH enzyme activity [80]. Forced expression of PDP2 into naïve CD4^+^ cells reduce Th17 cell differentiation [80]. These data demonstrate that molecules which were previously connected to T cell effector function accomplish their effects by directly controlling the expression of distinct enzymes involved in cell metabolism.

Because Tfh cells are also involved in the pathogenesis of SLE and their numbers are expanded, in vivo treatment of several lupus-prone mice with 2-DG normalized Tfh cells numbers and reversed serological markers of lupus but more importantly it did not affect humoral responses that preferentially relied on glutaminolysis [82,83]. This observation is of paramount importance because it points to the need to understand the differential regulation of metabolic pathways between the development of a normal and an autoimmune/inflammatory process.

Compared to CD4^+^ T cells, stimulated cytotoxic CD8^+^ cells undergo more rapid growth and proliferation and retain preferential glycolytic metabolism resistant to metabolic inhibition [58]. CD38 is ecto-enzyme NADase, a co-factor of OXPHOS, found to be overexpressed on SLE T cell subsets [84,85]. In vitro generated T cells lacking CD38 have enhanced oxidative phosphorylation and higher glutaminolysis rates [86]. Recently we found that CD8^+^CD38^high^ population is expanded in subset of patients with SLE who have increased rates of infections and these cells had decreased cytotoxic capacity, degranulation and expression of cytolytic enzymes [87]. These findings point to the need to develop biologics or drugs to inhibit CD38 in order to restore CD8^+^ cytotoxic T cell responses and avert infections, which are still the main cause of mortality in people with SLE.

## GLUTAMINE METABOLISM

Glutamine is a non-essential amino acid and another important metabolic fuel besides glucose. Glutaminolysis has a vital role in energy production in proliferating cells, including T cells. Glutamine enters the cell through the alanine, serine, cysteine-preferring transporter 2 (ASCT2) and is converted to glutamate, which is further transformed into α-ketoglutarate, an intermediate of the TCA cycle. Glutaminolysis is requisite for mTORC activation [88] and for the generation of glutathione, which neutralized ROS and is essential for Th17 cell differentiation [89,90]. Glutamine metabolism is involved T cell differentiation and fate. Th17 cells depend on glutaminolysis more than Th1, Th2 and Treg cells [88]. Depletion of glutamine or deficiency of the transporter ASCT2 reduces Th1 and Th17 differentiation [91]. Glutaminase which generates glutamate from glutamine has two isoforms: kidney-type glutaminase 1 and livertype glutaminase 2. Glutaminase 1 has more enzymatic activity than glutaminase 2 and T cells express mainly glutaminase 1 [88]. The transcription factor ICER binds the promoter lesion of glutaminase 1 and enhances its expression and promotes glutaminolysis [88] (Figure 1). Inhibition of glutaminase 1 or deficiency of glutaminase 1 reduces Th17 cell differentiation [88,92] and disease activity in animals subjected to experimental autoimmune encephalomyelitis (EAE). The glutaminase inhibitor, Bis-2-(5-phenylacetamido-1,3,4-thiadiazol-2-yl)ethyl sulfide (BPTES)], also ameliorates the disease activity in MRL*/lpr* mice [93] (Table 1).

Glutamate can generate α-ketoglutarate through direct deamination by glutamate dehydrogenase or through transamination to produce the non-essential amino acid alanine or aspartate. Glutamate oxaloacetate transaminase 1 (GOT1) catalyzes the conversion of glutamate to α-ketoglutarate via the transamination of oxaloacetate to aspartate. Selective inhibition of GOT1 with (aminooxy)acetic acid (AOA) reduces Th17 differentiation and enhances Treg cells differentiation and ameliorates EAE [94].

Tfh cells are increased in both the patients with SLE and lupus-prone mice and their numbers correlate with disease activity. Glutaminolysis also regulates Tfh and inhibition of glutaminolysis with the glutamine analog 6-Diazo-5-oxo-L-norleucine (DON) reduces the frequency of Tfh cells and the production of dsDNA antibody [83].

## LIPID METABOLISM

Fatty acid oxidation (FAO) is a mitochondrial aerobic process responsible for producing acetyl CoA from fatty acids which enters the TCA cycle (Figure 1). Quiescent T cells and Treg cells use mainly FAO. The addition of fatty acids to cells in culture increases Treg cell, but not effector T cell differentiation [95]. Adenosine monophosphate activated protein kinase (AMPK) is serine/threonine kinase and one of the key metabolic regulators besides mTORC. AMPK inhibits mTORC activity and vice versa. AMPK increases the expression of carnitine palmitoyl transferase I (CPT I), a rate-limiting enzyme in FAO and promotes FAO, whereas AMPK-dependent phosphorylation of acetyl-CoA carboxylase 1 (ACC1) inhibits fatty acid synthesis [96,97]. In fact, Treg cells have high expression levels of CPT I, which supports Treg cells to use multiple fuel sources, including FAO [59,98].

Biosynthesis of fatty acids and cholesterol is essential for T cell proliferation, and differentiation in effector T cells, especially Th17 cells. Fatty acid synthesis is a cytosolic process whereby acetyl CoA is converted to fatty acids. ACC1, the rate-limiting enzyme for fatty acid synthesis promotes metabolic reprograming due to TCR stimulation, and enhances Th1 and Th17 cell differentiation [61,99,100]. Cholesterol is synthesized from acetyl CoA by the hydroxymethylglutaryl-coenzyme A (HMG-CoA). Statin, the inhibitors of HMG-CoA reductase, reduce Th17 cell differentiation [101].

Lipid rafts are subdomains of the plasma membrane that are composed of cholesterol and glycosphingolipids. CD4^+^ T cells from people with SLE have an altered profile of lipid raft–associated glycosphingolipids compared with that of healthy controls [102]. *N*-butyldeoxynojirimycin (NB-DNJ), a glucosylceramide synthase inhibitor, normalizes lipid metabolism in CD4^+^ T cells from the patients with SLE [102]. Furthermore, NB-DNJ treatment restores the functionality of B and T lymphocyte attenuator (BTLA), an inhibitory receptor, similar to CTLA-4 and PD-1, in lupus CD4^+^ T cells [103] (Table 1). The synthesis of glycosphingolipids in T, B cells and kidney is regulated by the transcription factor Friend leukaemia integration 1 (FLI1). A polymorphic microsatellite consisting of GA repeats within the proximal promoter of *Fli1* gene is shorter in three different lupus-prone mice, and the length of the microsatellite correlates inversely with the activity of the promoter [104]. Overexpression of FLI1 in mice results in a progressive immunological renal disease and renal failure caused by tubulointerstitial nephritis and immune-complex glomerulonephritis [105]. *Fli1*^+/−^ T cells from MRL/*lpr* mice transferred to *Rag1*-deficient mice have reduced levels of glycosphingolipids and diminished TCR activation compared with transferred *Fli1*^+/+^ T cells [106]. The formation of lipid rafts on the surface of T cells is important during T cell activation and T cells from people with SLE aggregate lipid rafts on the surface membrane spontaneously [105] and enable faster and stronger CD3-mediated cell signaling. Enhanced lipid raft aggregation in the absence of obvious antigenic stimulation implies that the surface membrane is more fluid and molecules move around faster. There is need to gain more information on metabolic factors that regulate the expression of lipids on the surface membrane of T cells so we may control their signaling capacity.

## CONCLUSIONS AND FUTURE DIRECTIONS

We have discussed in detail most recent information on the metabolic aberrations which account for the abnormal function of T cell subsets in people with SLE. We have learned that specific effector T cell function is defined by metabolic processes which dictate the sources of energy generation. More importantly, we have learned that molecules such as kinases (CaMK4) or transcription factors (CREM/ICER) which had previously been linked to abnormal effector T cell function in SLE accomplish their effects by directly controlling the function of metabolic enzymes involved in glycolysis and glutaminolysis. It is certain that in the near future we will discover that other known determinants of effector T cell function accomplish their effect through the control of metabolic enzymes. Therefore, it is proper to assume that what each T cell does depends on its source and disposal of energy. Besides though energy, each metabolic pathway generates metabolites which are important to construct molecules needed in other cells processes including building blocks for cell growth and differentiation. It is important to consider that metabolic processes may behave differently during the development of a normal immune response and in the context of autoimmune or inflammatory context. Such understanding should influence the design of approaches to boost a normal response and suppress an inflammatory one. We expect that modulators of metabolic processes will be important in controlling abnormal T cell behavior and although most probably they alone will not be sufficient to control autoimmune pathology, they may be perfect adjuvants to standard treatment with immunosuppressive drugs and help limit their side effects by decreasing their dose. Finally, we should state unequivocally, that more research is needed to completely understand the complex metabolic processes that are responsible for the well-known aberrant function of T cell subsets including Treg, CD8^+^ cytotoxic, T effector and T follicular helper cells. Very little, if anything, is known on the metabolism of lipids in SLE immune cells. For example, does cholesterol control immune cell function, and does cholesterol of fatty acids control immune cell membrane physical chemistry behavior. For example, what accounts for the spontaneous formation of lipid rafts on the surface membrane of T cells. It is plausible that aberrant lipid/sphingolipid metabolism contributes to their formation and indirectly to the enhanced early signaling events [22].

## Figures and Tables

**Table 1.** Potential therapeutic target of metabolic pathway in SLE.

Therapeutic target	Therapy	Effect on T cells	Effects on SLE
Hexokinase and mitochondrial complex I	2-deoxy-d glucose and metformin	Decrease IFNγ production and decreases Tfh cells	Reduces disease activity, and improve kidney disease
Glutaminase 1	BPTES, CB-839, and 968	Reduces Th17 cell differentiation	Reduces disease activity, and improve kidney disease
Mitochondrial metabolism	Bz-423	Promotes autoreactive T cell apoptosis	Reduces disease activity
Glucosylceramide synthetase	NB-DNJ	Normalizes TCR signaling and restores BTLA expression	Reduces disease activity
Cysteine metabolism	*N*-acetyl cysteine	Inhibits mTOR activity	Reduces disease activity, and improve kidney disease
mTOR signaling	Sirolimus	Inhibits Th17 differentiation and promotes Treg differentiation	Reduces disease activity
PPARγ	Pioglitazone (agonist)	Promotes Treg expansion	Improves nephritis

BTLA, B and T lymphocyte attenuator; BPTES, bis-2-(5-phenylacetamido-1,3,4-thiadiazol-2-yl)ethyl sulfide; mTOR, mammalian target of rapamycin; PPARγ, peroxisome proliferator-activated receptor γ; Tfh, follicular helper T cells.

## ABBREVIATIONS

Acetyl CoA: acetyl coenzyme A
AOA: (aminooxy)acetic acid
ATP: adenosine triphosphate
ASCT2: alanine, serine, cysteine-preferring transporter 2
BTLA: B and T lymphocyte attenuator
BPTES: bis-2-(5-phenylacetamido-1,3,4-thiadiazol-2-yl)ethyl sulfide
CaMK4: calcium/calmodulin–dependent protein kinase IV
CRE: cAMP-response element
CTLA: cytotoxic T lymphocyte–associated protein 4
CREM: cAMP response element modulator
DON: 6-diazo-5-oxo-L-norleucine
EAE: experimental autoimmune encephalomyelitis
FAO: fatty acid oxidation
GOT: glutamate oxaloacetate transaminase
HIF: hypoxia-inducible factor
HMG-CoA: hydroxymethylglutaryl-coenzyme A
ICER: inducible cAMP early repressor
IL: interleukin
mTOR: mammalian target of rapamycin
mTORC: mammalian target of rapamycin complex
OXPHOS: oxidative phosphorylation
PDH: pyruvate dehydrogenase
PDP: pyruvate dehydrogenase phosphatase catalytic subunit
PD-1: programmed death 1
PPP: pentose phosphate pathway
PP2A: phosphatase2A
RORγt: retinoic acid-related orphan receptor gamma t
ROS: reactive oxygen species
SLE: systemic lupus erythematosus
STAT3: signal transducer and activator of transcription 3
TCA: tricarboxylic acid
TCR: T-cell receptor
Tfh: follicular helper T cells
Treg: regulatory T cell
2-DG: 2-deoxy-D glucose
